# Supplementary material for: A transcription factor collective defines the HSN serotonergic neuron regulatory landscape
Source: eLife. 2018 Mar 22;7:e32785. doi: 10.7554/eLife.32785 (PMC5916565; doi:10.7554/eLife.32785)
Supplement: Supplementary file 3. — Related to Materials and methods. [file elife-32785-supp3.docx]

**Supplementary File 3. Worm strains**

| **Strain** | **Genotype** |
| --- | --- |
| AL132 | *icIs132(unc-40::gfp)* |
| BC13535 | *sIs13247(nlg-1::gfp)* |
| BL5717 | *inIs179(ida-1::gfp)II* |
| BL5752 | *inIs181(ida-1::gfp); inIs182(ida-1::gfp)* |
| GR1333 | *yzIs71[tph-1::gfp, rol-6(su1006)]V* |
| LX1376 | *vsEx580[kcc-2c::gfp, myo-2::gfp]* |
| MH1337 | *kuIs34(sem-4::gfp)IV* |
| MH1346 | *kuIs35(sem-4::gfp)* |
| MT1069 | *egl-18(n474)IV* + anti 5-HT |
| MT1570 | *egl-18(n475)IV +* anti 5-HT |
| N2 | N2 |
| NFB1025 | *egl-18(ok290)IV; vlcEx1[tph-1prom2::gfp (50ng/ul), rol-6(su1006)]* |
| NFB1026 | *egl-18(ok290)IV; otIs291[rab-3::gfp, rol-6(su1006)]* |
| NFB1027 | *egl-18(ok290)IV; vlcEx279[cat-1prom14::gfp (50ng/ul),rol-6(su1006)]* |
| NFB1029 | *egl-18(ok290)IV; vsEx580[kcc-2c::gfp, myo-2::gfp]* |
| NFB1031 | *hlh-3(tm1688)II; egl-46(sy628)V; otIs226(bas-1::gfp)IV* |
| NFB1033 | *egl-18(ok290)IV; egl-46(sy628)V; otIs221(cat-1::gfp)III* |
| NFB1079 | *vlcEx588[aak-2p::GFP::unc-54 3'UTR (50ng/ul), rol-6(su1006)* |
| NFB1090 | *vlcEx599[ast-1 w12::GFP::unc-54 3'UTR (50ng/ul), rol-6(su1006) (100ng/ul)]* |
| NFB11 | *ast-1(hd1), rol-6(su1006)II; zdIs13(tph-1::gfp)IV; him-5* |
| NFB1103 | *vlcEx609(bas-1prom13::gfp (50ng/ul, rol-6))* |
| NFB1110 | *vlcEx614(tph-1 prom60::gfp (50ng/ul), rol-6)* |
| NFB1139 | *vlcEx641 [F37A8.5 w31::GFP::unc-54 3'UTR (50ng/ul), rol-6(su1006) (100ng/ul)]* |
| NFB1149 | *vlcEx651 [lgc-49 w7::GFP::unc-54 3'UTR (50ng/ul), rol-6(su1006) (100ng/ul)]* |
| NFB1151 | *vlcEx653 [pde-3 w2-3::GFP::unc-54 3'UTR (50ng/ul), rol-6(su1006) (100ng/ul)]* |
| NFB1164 | *vlcEx666 [klp-7 w5-6::GFP::unc-54 3'UTR (50ng/ul), rol-6(su1006) (100ng/ul)]* |
| NFB117 | *vlcEx16(bas-1prom18::gfp(50ng/ul), rol-6)* |
| NFB1171 | *vlcEx673 [unc-32 w4-6::GFP::unc-54 3'UTR (50ng/ul), rol-6(su1006) (100ng/ul)]* |
| NFB1181 | *vlcEx683 [mgl-2* *w2-4::GFP::unc-54 3'UTR (50ng/ul), rol-6(su1006) (100ng/ul)]* |
| NFB1187 | *unc-86(n848)III; egl-46(sy628)V; otIs226(bas-1::gfp)IV* |
| NFB1215 | *vlcEx709 [mec-10 w1-4::GFP::unc-54 3'UTR (50ng/ul), rol-6(su1006) (100ng/ul)]* |
| NFB1218 | *vlcEx712 [npr-1 w6-12::GFP::unc-54 3'UTR (50ng/ul), rol-6(su1006) (100ng/ul)]* |
| NFB1300 | *egl-18(ok290)IV; kuIs35(sem-4::gfp)* |
| NFB1352 | *vlcEx800[pan-1 w1-2::GFP::unc-54 3'UTR (50ng/ul), rol-6(su1006) (100ng/ul)]* |
| NFB1369 | *ast-1(vlc19[ast-1::gfp])II* |
| NFB1375 | *unc-86(n846)III; ast-1(vlc19[ast-1::gfp])II* |
| NFB1377 | *hlh-3(tm1688)II; ast-1(vlc19[ast-1::gfp])II* |
| NFB1379 | *egl-46(sy628)V; ast-1(vlc19[ast-1::gfp])II* |
| NFB1381 | *sem-4(n1971)I; ast-1(vlc19[ast-1::gfp])II* |
| NFB1383 | *egl-18(ok290)IV; ast-1(vlc19[ast-1::gfp])II* |
| NFB1388 | *egl-46(gk692)V; zdIs13(tph-1::gfp)IV* |
| NFB1389 | *egl-46(gk692)V; otIs221(cat-1::gfp)III* |
| NFB1390 | *egl-46(gk692)V; otIs226(bas-1::gfp)IV* |
| NFB1391 | *egl-46(gk692)V; otIs225(cat-4)II* |
| NFB1395 | *egl-46(gk692)V; nIs34(flp-19::gfp)* |
| NFB1468 | *egl-18(ok290)IV; inIs179(ida-1::gfp)II* |
| NFB1469 | *egl-18(ok290), otIs33(kal-1::gfp)IV* |
| NFB152 | *sem-4(n1971)I; vsEx580[kcc-2c::gfp, myo-2::gfp]* |
| NFB153 | *unc-86(n846)III; vsEx580[kcc-2c::gfp, myo-2::gfp]* |
| NFB154 | *sem-4(n1971)I; otIs287[rab-3::yfp, rol-6(su1006)]IV* |
| NFB155 | *sem-4(n1971)I; inIs179(ida-1::gfp)II* |
| NFB156 | *sem-4(n1971)I; zfIs6(lgc-55::gfp)II* |
| NFB157 | *sem-4(n1971)I; ynIs34(flp-19::gfp) IV; him-5(e1490)V* |
| NFB158 | *unc-86(n846)III; sIs13247(nlg-1::gfp)* |
| NFB159 | *ast-1(hd92); vlcEx844[ast-1(+), cat-1::DsRed]; otIs226(bas-1::gfp)IV* |
| NFB160 | *ast-1(hd92); vlcEx845[ast-1(+), cat-1::DsRed]; otIs226(bas-1::gfp)IV* |
| NFB1691 | *zdIs13(tph-1::gfp)IV; vlcEx1020[HSNearlyprom::ast-1, rol-6(su1006)]* |
| NFB1692 | *hlh-3(vlc28[hlh-3::neongreen])II* |
| NFB1732 | *sem-4(n1971)I; hlh-3(vlc28[hlh-3::mNeonGreen])II* |
| NFB1733 | *unc-86(n846)III; hlh-3(vlc28[hlh-3::mNeonGreen])II* |
| NFB1734 | *egl-46(sy628)V; hlh-3(vlc28[hlh-3::mNeonGreen])II* |
| NFB1735 | *egl-18(ok290)IV; hlh-3(vlc28[hlh-3::mNeonGreen])II* |
| NFB1737 | *ast-1(ot417), hlh-3(vlc28[hlh-3::mNeonGreen])II* |
| NFB1745 | *zdIs13(tph-1::gfp)IV; vlcEx1048[hsp16-2::hlh-3; HSNearlyprom::ast-1; ttx-3::mCherry; rol-6(su10006)]* |
| NFB1747 | *zdIs13(tph-1::gfp)IV; vlcEx1050[hsp16-2::hlh-3; HSNearlyprom::ast-1; ttx-3::mCherry; rol-6(su10006)]* |
| NFB1757 | *egl-18(ok290)IV; yzIs71[tph-1::gfp, rol-6(su1006)]V; vlcEx1051[c4p8::Gata2 (50ngµl), ttx-3::mCherry (50ngµl), rol6(su1006) (50ngµl)]* |
| NFB1758 | *egl-18(ok290)IV; yzIs71[tph-1::gfp, rol-6(su1006)]V; vlcEx1052[c4p8::Gata2 (50ngµl), ttx-3::mCherry (50ngµl), rol6(su1006) (50ngµl)]* |
| NFB178 | *unc-86(n846)III; icIs132 (unc-40::gfp); him-8(e1489)IV* |
| NFB179 | *sem-4(n1971)I; icIs132 (unc-40::gfp); him-8(e1489)IV* |
| NFB1817 | *egl-46(sy628)V; vlcEx2(tph-1 prom2::gfp (50ng/ul), rol-6)* |
| NFB1818 | *hlh-3 (tm1688) II; vlcEx302(cat-1prom76::gfp (50ng/ul),rol-6)* |
| NFB1819 | *sem-4(n2654)I; vlcEx302(cat-1prom76::gfp (50ng/ul),rol-6)* |
| NFB1820 | *egl-46(sy628)V; vlcEx609(bas-1prom13::gfp (50ng/ul, rol-6))* |
| NFB1821 | *ast-1(ot417)II;vlcEx145(bas-1prom65::gfp (50ng/ul),rol-6)* |
| NFB1854 | *ast-1(ot417) II; vlcEx16(bas-1prom18::gfp(50ng/ul), rol-6)* |
| NFB1855 | *egl-46(sy628)V; vlcEx614(tph-1 prom60::gfp (50ng/ul), rol-6)* |
| NFB1856 | *egl-46(sy628)V; vlcEx354(bas-1prom77::gfp (50ng/ul),rol-6)* |
| NFB186 | *sem-4(n1971)I; otIs33(kal-1::gfp)IV* |
| NFB187 | *ast-1(ot417)II; zfIs4(lgc-55::mCherry)* |
| NFB1870 | *zdIs13(tph-1::gfp)IV; vlcEx1082[HSNearlyprom::ast-1 (50ng/ul), ttx-3::mcherry (50ng/ul), rol-6(su10006) (50ng/ul)]* |
| NFB1871 | *zdIs13(tph-1::gfp)IV; vlcEx1083[HSNearlyprom::ast-1 (50ng/ul), ttx-3::mcherry (50ng/ul), rol-6(su10006) (50ng/ul)]* |
| NFB1873 | *zdIs13(tph-1::gfp)IV; vlcEx1085[HSNearlyprom::hlh-3 (50ng/ul), ttx-3::mcherry (50ng/ul), rol-6(su10006) (50ng/ul)]* |
| NFB1874 | *zdIs13(tph-1::gfp)IV; vlcEx1086[HSNearlyprom::hlh-3 (50ng/ul), ttx-3::mcherry (50ng/ul), rol-6(su10006) (50ng/ul)]* |
| NFB1876 | *zdIs13(tph-1::gfp)IV; vlcEx1088[HSNearlyprom::hlh-3 (50ng/ul), HSNearlyprom::ast-1 (50ng/ul), ttx-3::mcherry (25ng/ul), rol-6(su10006) (25ng/ul)]* |
| NFB1877 | *zdIs13(tph-1::gfp)IV; vlcEx1089[HSNearlyprom::hlh-3 (50ng/ul), HSNearlyprom::ast-1 (50ng/ul), ttx-3::mcherry (25ng/ul), rol-6(su10006) (25ng/ul)]* |
| NFB1884 | *unc-86(n846)III; vlcEx653 [pde-3 w2-3::GFP::unc-54 3'UTR (50ng/ul), rol-6(su1006) (100ng/ul)]* |
| NFB1885 | *unc-86(n846)III; vlcEx683 [mgl-2 w2-4::GFP::unc-54 3'UTR (50ng/ul), rol-6(su1006) (100ng/ul)]* |
| NFB1886 | *unc-86(n846)III; vlcEx709 [mec-10 w1-4::GFP::unc-54 3'UTR (50ng/ul), rol-6(su1006) (100ng/ul)]* |
| NFB1887 | *unc-86(n846)III; vlcEx712 [npr-1 w6-12::GFP::unc-54 3'UTR (50ng/ul), rol-6(su1006) (100ng/ul)]* |
| NFB1888 | *unc-86(n846)III; vlcEx599[ast-1 w12::GFP::unc-54 3'UTR (50ng/ul), rol-6(su1006) (100ng/ul)]* |
| NFB1889 | *unc-86(n846)III; vlcEx651 [lgc-49 w7::GFP::unc-54 3'UTR (50ng/ul), rol-6(su1006) (100ng/ul)]* |
| NFB189 | *sem-4(n1971)I; otIs337(unc-86fosmid::NLS::YFP::H2B, ttx-3::mCherry)* |
| NFB1890 | *unc-86(n846)III; vlcEx673 [unc-32 w4-6::GFP::unc-54 3'UTR (50ng/ul), rol-6(su1006) (100ng/ul)]* |
| NFB1891 | *unc-86(n846)III; zdIs13(tph-1::gfp)IV; vlcEx641 [F37A8.5 w31::GFP::unc-54 3'UTR (50ng/ul), rol-6(su1006) (100ng/ul)]* |
| NFB1892 | *unc-86(n846)III; vlcEx588[aak-2p::GFP::unc-54 3'UTR (50ng/ul), rol-6(su1006)* |
| NFB1893 | *unc-86(n846)III; vlcEx800[pan-1 w1-2::GFP::unc-54 3'UTR (50ng/ul), rol-6(su1006) (100ng/ul)]* |
| NFB1894 | *unc-86(n846)III; vlcEx666 [klp-7 w5-6::GFP::unc-54 3'UTR (50ng/ul), rol-6(su1006) (100ng/ul)]* |
| NFB1895 | *vlcEx1091[snt-1* *w2-9::GFP::unc-54 3'UTR (50ng/ul), rol-6(su1006) (100ng/ul)]* |
| NFB1896 | *unc-86(n846)III; vlcEx1091 [snt-1 w2-9::GFP::unc-54 3'UTR (50ng/ul), rol-6(su1006) (100ng/ul)]* |
| NFB1897 | *vlcEx1092 [sprr-1 w29::GFP::unc-54 3'UTR (50ng/ul), rol-6(su1006) (100ng/ul)]* |
| NFB1898 | *unc-86(n846)III; vlcEx1092 [sprr-1 w29::GFP::unc-54 3'UTR (50ng/ul), rol-6(su1006) (100ng/ul)]* |
| NFB1899 | *vlcEx1093 [sem-4 w18-23::GFP::unc-54 3'UTR (50ng/ul), rol-6(su1006) (100ng/ul)]* |
| NFB190 | *unc-86(n846)III; kuIs34(sem-4::gfp)IV* |
| NFB1900 | *unc-86(n846)III; vlcEx1093 [sem-4 w18-23::GFP::unc-54 3'UTR (50ng/ul), rol-6(su1006) (100ng/ul)]* |
| NFB1901 | *vlcEx1094 [C53B4.4* *w2-10::GFP::unc-54 3'UTR (50ng/ul), rol-6(su1006) (100ng/ul)]* |
| NFB1902 | *unc-86(n846)III; vlcEx1094 [C53B4.4 w2-10::GFP::unc-54 3'UTR (50ng/ul), rol-6(su1006) (100ng/ul)]* |
| NFB1905 | *hlh-3(ot354)II; otIs221(cat-1::gfp)III* |
| NFB1906 | *hlh-3(ot354)II; zdIs13(tph-1::gfp)IV* |
| NFB1907 | *egl-18(n474)IV; otIs221(cat-1::gfp)III* |
| NFB1908 | *egl-18(n475)IV; otIs221(cat-1::gfp)III* |
| NFB1909 | *egl-18(n474)IV; yzIs71[(tph-1::gfp, rol-6(su10006)]V* |
| NFB1910 | *egl-18(n475)IV; yzIs71[(tph-1::gfp, rol-6(su10006)]V* |
| NFB250 | *sem-4(n1971)I; sIs13247(nlg-1::gfp)* |
| NFB251 | *ast-1(ot417)II; sIs13247(nlg-1::gfp)* |
| NFB252 | *ast-1(ot417)II; icIs132 (unc-40::gfp); him-8(e1489)IV* |
| NFB253 | *ast-1(ot417)II; unc-86(n848)III; otIs226[bas-1::gfp]IV* |
| NFB259 | *ast-1(ot417)II; inIs181(ida-1::gfp); inIs182(ida-1::gfp)* |
| NFB282 | *vlcEx145(bas-1prom65::gfp (50ng/ul),rol-6)* |
| NFB290 | *ast-1(ot417)II; zdIs13(tph-1::gfp)IV; vlcEx148[bas-1prom::ast-1 (5ngµl), ttx-3::mCherry (50ngµl); rol-6(su1006) (50ngµl)]* |
| NFB336 | *ast-1(ot417)II; zdIs13(tph-1::gfp)IV; vlcEx148[bas-1prom::Pet1 (10ngµl), ttx-3::mCherry (50ngµl); rol-6(su1006) (50ngµl)]* |
| NFB38 | *unc-86(n846)III; ynIs34(flp-19::gfp) IV; him-5(e1490)V* |
| NFB39 | *ast-1(ot417)II; ynIs34(flp-19::gfp)IV; him-5(e1490)V* |
| NFB395 | *sem-4(n2654)I; ast-1(ot417)II; zdIs13(tph-1::gfp)IV* |
| NFB411 | *vlcEx236(cat-1prom71::gfp (50ng/ul),rol-6)* |
| NFB417 | *sem-4(n2654)I; zdIs13(tph-1::gfp)IV* |
| NFB419 | *egl-46(sy628)V; stIs11606[egl-18a::H1-WCHERRY + unc-119(+)]* |
| NFB42 | *unc-86(n846)III; inIs179(ida-1::gfp)II* |
| NFB430 | *sem-4(n1971)I; stIs11606[egl-18a::H1-WCHERRY + unc-119(+)]* |
| NFB44 | *unc-86(n846)III; otIs33(kal-1::gfp)IV* |
| NFB448 | *unc-86(n846)III; zfIs6(lgc-55::gfp)II* |
| NFB45 | *ast-1(ot417)II; otIs33(kal-1::gfp)IV* |
| NFB450 | *sem-4(n2654)I; otIs226(bas-1::gfp)IV* |
| NFB451 | *sem-4(n2654)I; otIs221(cat-1::gfp)III* |
| NFB453 | *egl-46(sy628)V; icIs132 (unc-40::gfp); him-8(e1489)IV* |
| NFB455 | *hlh-3(tm1688)II; otIs226(bas-1::gfp)IV* |
| NFB46 | *ast-1(hd92)II; otIs221(cat-1::gfp)III* |
| NFB47 | *ast-1(ot417)II; vsEx580[kcc-2c::gfp, myo-2::gfp]* |
| NFB471 | *hlh-3(tm1688)II; zdIs13(tph-1::gfp)IV* |
| NFB472 | *hlh-3(tm1688)II; otEx2470[cat-4::gfp (50ng/ul), rol-6(su1006)]* |
| NFB473 | *hlh-3(tm1688)II; zfIs4(lgc-55::mCherry)* |
| NFB474 | *hlh-3(tm1688)II; otIs287[rab-3::yfp, rol-6(su1006)]IV* |
| NFB475 | *hlh-3(tm1688)II; otIs33(kal-1::gfp)IV* |
| NFB476 | *hlh-3(tm1688)II; otIs337(unc-86fosmid::NLS::YFP::H2B, ttx-3::mCherry)* |
| NFB477 | *egl-46(sy628)V; zdIs13(tph-1::gfp)IV* |
| NFB478 | *egl-46(sy628)V; otIs221(cat-1::gfp)III* |
| NFB479 | *egl-46(sy628)V; otIs225(cat-4::gfp)II* |
| NFB480 | *egl-46(sy628)V; otIs33 (kal-1::gfp)IV* |
| NFB481 | *egl-46(sy628)V; otIs337(unc-86fosmid::NLS::YFP::H2B, ttx-3::mCherry)* |
| NFB486 | *hlh-3(tm1688)II; ynIs34(flp-19::gfp) IV* |
| NFB489 | *hlh-3(tm1688)II; otIs221(cat-1::gfp)III* |
| NFB503 | *vlcEx279(cat-1prom14::gfp (50ng/ul),rol-6)* |
| NFB509 | *zdIs13(tph-1::gfp)IV; vlcEx284 [hsp16-2:hlh-3; ttx-3::mCherry; rol-6(su10006)]* |
| NFB510 | *hlh-3(tm1688)II; vsEx580[kcc-2c::gfp, myo-2::gfp]* |
| NFB511 | *egl-46(sy628)V; vsEx580[kcc-2c::gfp, myo-2::gfp]* |
| NFB516 | *hlh-3(tm1688)II; kuIs34(sem-4::gfp)IV* |
| NFB517 | *hlh-3(tm1688)II; sIs13247(nlg-1::gfp)* |
| NFB518 | *egl-46(sy628)V; ynIs34(flp-19::gfp) IV* |
| NFB525 | *egl-46(sy628)V; kuIs34(sem-4::gfp)IV* |
| NFB526 | *egl-46(sy628)V; zfIs6(lgc-55::gfp)II* |
| NFB536 | *egl-46(sy628)V; otIs226(bas-1::gfp)IV* |
| NFB537 | *egl-46(sy628)V; otIs287[rab-3::yfp, rol-6(su1006)]IV* |
| NFB538 | *egl-46(sy628)V; inIs179(ida-1::gfp)II* |
| NFB539 | *hlh-3(tm1688)II; inIs181(ida-1::gfp); inIs182(ida-1::gfp)* |
| NFB552 | *hlh-3(tm1688)II; icIs132 (unc-40::gfp); him-8(e1489)IV* |
| NFB557 | *vlcEx302(cat-1prom76::gfp (50ng/ul),rol-6)* |
| NFB6 | *zdIs13(tph-1::gfp)IV; otIs198(hsp16-2::ast-1, hsp16-2::NLS::mCherry, ttx-3::dsRed)* |
| NFB603 | *unc-86(n848)III; otIs224(cat-1::gfp)V* |
| NFB605 | *sem-4(n2654)I; ast-1(ot417)II; otIs226(bas-1::gfp)IV* |
| NFB607 | *hlh-3(tm1688)II; stIs11606[egl-18a::H1-WCHERRY + unc-119(+)]* |
| NFB608 | *vlcEx324[egl-46prom::dsRed; ttx-3::mCherry; rol-6(su1006)]* |
| NFB62 | *ast-1(ot417)II; otIs337(unc-86fosmid::NLS::YFP::H2B, ttx-3::mCherry)* |
| NFB624 | *zdIs13(tph-1::gfp)IV; vlcEx334[hsp16-2::hlh-3; ttx-3::mCherry; rol-6(su10006)]* |
| NFB63 | *ast-1(ot417)II; otIs287[rab-3::yfp, rol-6(su1006)]IV* |
| NFB633 | *egl-46(sy628)V; sIs13247(nlg-1::gfp)* |
| NFB645 | *sem-4(n2654)I; otIs224(cat-1::gfp)V* |
| NFB647 | *hlh-3(tm1688)II; otIs224(cat-1::gfp)V* |
| NFB648 | *sem-4(n2654)I; unc-86(n848)III; otIs224(cat-1::gfp)V* |
| NFB649 | *sem-4(n1971)I; zdIs13(tph-1::gfp)IV; vlcEx511[kal-1prom::Sall2 (20ngµl),, ttx-3::mCherry (50ngµl), rol-6(su1006) (50ngµl)]* |
| NFB650 | *hlh-3(tm1688)II; unc-86(n848)III; otIs224(cat-1::gfp)V* |
| NFB651 | *hlh-3(tm1688)II; vlcEx324[egl-46prom::dsRed; ttx-3::mCherry; rol-6(su1006)]* |
| NFB652 | *unc-86(n846)III; vlcEx324[egl-46prom::dsRed; ttx-3::mCherry; rol-6(su1006)]* |
| NFB663 | *vlcEx354(bas-1prom77::gfp (50ng/ul),rol-6)* |
| NFB685 | *egl-18(ok290)IV; otIs225(cat-4::gfp)II* |
| NFB687 | *egl-18(ok290)IV; otIs221(cat-1::gfp)III* |
| NFB688 | *unc-86(n846)III; stIs11606[egl-18a::H1-WCHERRY + unc-119(+)]* |
| NFB689 | *rrf-3(pk1426)II; otIs517[(tph-1::SL2::YFP::H2B), ttx-3::mCherry, rol-6(su1006)]* |
| NFB695 | *ast-1(ot417)II; egl-46(sy628)V; otIs226(bas-1::gfp)IV* |
| NFB71 | *vlcEx2(tph-1 prom2::gfp (50ng/ul), rol-6)* |
| NFB711 | *vlcEx382(bas-1prom78::gfp (50ng/ul),rol-6)* |
| NFB715 | *egl-18(ok290)IV; otex2435[bas-1::gfp (50ng/ul), rol-6(su1006]* |
| NFB717 | *ast-1(ot417)II; vlcEx324[egl-46prom::dsRed; ttx-3::mCherry; rol-6(su1006)]* |
| NFB720 | *sem-4(n1971)I; vlcEx324[egl-46prom::dsRed; ttx-3::mCherry; rol-6(su1006)]* |
| NFB735 | *ast-1(ot417)II; vlcEx16[bas-1prom18::gfp(50ng/ul), rol-6(su1006)]* |
| NFB745 | *rrf-3(pk1426)II; vlcEx400[(cat-1::MDM2::gfp (100 ng/ul), ttx-3::mCherry (30ng/ul), rol-6(su1006)]* |
| NFB754 | *ast-1(ot417)II; stIs11606[egl-18a::H1-WCHERRY + unc-119(+)]* |
| NFB755 | *egl-18(ok290)IV; sem-4(n2654)I; otIs221(cat-1::gfp)III* |
| NFB756 | *egl-18(ok290)IV; sem-4(n2654)I; zdIs13(tph-1::gfp)IV* |
| NFB757 | *egl-46(sy628)V; otIs576(unc-17fosmid::GFP, lin-44::YFP)* |
| NFB827 | *hlh-3(tm1688)II; vlcEx279(cat-1prom14::gfp (50ng/ul),rol-6)* |
| NFB83 | *ast-1(ot417)II; otEx2470[cat-4::gfp (50ng/ul), rol-6(su1006)]* |
| NFB837 | *ast-1(ot417)II;vlcEx382(bas-1prom78::gfp (50ng/ul),rol-6)* |
| NFB843 | *egl-18(ok290)IV; otIs337(unc-86fosmid::NLS::YFP::H2B, ttx-3::mCherry)* |
| NFB844 | *ast-1(ot417)II; otIs576(unc-17fosmid::GFP, lin-44::YFP)* |
| NFB855 | *unc-86(n846)III; otIs576(unc-17fosmid::GFP, lin-44::YFP); him-5(e1490)V* |
| NFB857 | *hlh-3(tm1688)II; otIs576(unc-17fosmid::GFP, lin-44::YFP); him-5(e1490)V* |
| NFB858 | *egl-18(ok290)IV; otIs576(unc-17fosmid::GFP, lin-44::YFP)* |
| NFB867 | *unc-86(n848)III; vlcEx279(cat-1prom14::gfp (50ng/ul),rol-6)* |
| NFB868 | *unc-86(n848)III; vlcEx236(cat-1prom71::gfp (50ng/ul),rol-6)* |
| NFB869 | *hlh-3(tm1688)II; vlcEx236(cat-1prom71::gfp (50ng/ul),rol-6)* |
| NFB870 | *sem-4(n2654)I; vlcEx279(cat-1prom14::gfp (50ng/ul),rol-6)* |
| NFB871 | *egl-18(ok290)IV;* *vlcEx324[egl-46prom::dsRed; ttx-3::mCherry; rol-6(su1006)]* |
| NFB891 | *sem-4(n1971)I; otIs576(unc-17fosmid::GFP, lin-44::YFP); him-5(e1490)V* |
| NFB901 | *sem-4(n2654)I; vlcEx236(cat-1prom71::gfp (50ng/ul),rol-6)* |
| NFB912 | *hlh-3(tm1688)II; zdIs13(tph-1::gfp)IV;* *vlcEx479[cat-4prom::Ascl1, ttx-3::mCherry, rol-6(su1006)]* |
| NFB913 | *hlh-3(tm1688)II; zdIs13(tph-1::gfp)IV;* *vlcEx480[cat-4prom::Ascl1(50ngµl), ttx-3::mCherry (50ngµl), rol-6(su1006) (50ngµl)]* |
| NFB914 | *egl-46(sy628)V; zdIs13(tph-1::gfp)IV; vlcEx481[cat-4prom::Inms1 (50ngµl), ttx-3::mCherry (50ngµl), rol-6(su1006) (50ngµl)]* |
| NFB931 | *hlh-3(tm1688)II; egl-18(ok290)IV; otex2435[bas-1::gfp(50ng/ul), rol-6(su1006)]* |
| NFB938 | *hlh-3(tm1688)II; zdIs13(tph-1::gfp)IV; vlcEx458[cat-4prom::hlh-3 (50ngµl), ttx-3::mCherry (50ngµl), rol-6(su1006) (50ngµl)]* |
| NFB939 | *egl-46(sy628)V; zdIs13(tph-1::gfp)IV;* *vlcEx471[cat-4prom::egl-46 (50ngµl), ttx-3::mCherry (50ngµl), rol-6(su1006) (50ngµl)]* |
| NFB940 | *egl-46(sy628)V; zdIs13(tph-1::gfp)IV;* *vlcEx472[cat-4prom::egl-46 (50ngµl), ttx-3::mCherry (50ngµl), rol-6(su1006) (50ngµl)]* |
| NFB962 | *ast-1(ot417)II; kuIs34(sem-4::gfp)IV* |
| NFB973 | *egl-18(ok290)IV; icIs132 (unc-40::gfp); him-8(e1489)IV* |
| NFB98 | *unc-86(n848)III; zdIs13(tph-1::gfp)IV* |
| NFB99 | *unc-86(n848)III; otIs226(bas-1::gfp)IV* |
| NFB996 | *egl-18(ok290)IV; zfIs6(lgc-55::gfp)II* |
| OH10425 | *otIs337(unc-86fosmid::NLS::YFP::H2B; ttx-3::mCherry)* |
| OH10562 | *ast-1(ot417)II; otIs226(bas-1::gfp)IV* |
| OH10603 | *unc-86(n846)III; otIs224(cat-1::gfp)V* |
| OH10607 | *unc-86(n846)III; otIs226(bas-1::gfp)IV* |
| OH10918 | *unc-86(n846)III; otIs225(cat-4::gfp)II* |
| OH11909 | *sem-4(n1971)I; otIs221(cat-1::gfp)III* |
| OH11910 | *sem-4(n1971)I; otIs226(bas-1::gfp)IV* |
| OH11962 | *sem-4(n1971)I; otIs225(cat-4::gfp)II* |
| OH11963 | *sem-4(n1971)I; zdIs13(tph-1::gfp)IV* |
| OH13083 | *otIs576(unc-17fosmid::GFP, lin-44::YFP)* |
| OH4196 | *otex2435[bas-1::gfp(50ng/ul), rol-6(su1006)]* |
| OH4255 | *otEx2470[cat-4::gfp(50ng/ul), rol-6(su1006)]* |
| OH8246 | *otIs221(cat-1::gfp)III* |
| OH8249 | *otIs224(cat-1::gfp)V* |
| OH8250 | *otIs225(cat-4::gfp)II* |
| OH8251 | *otIs226(bas-1::gfp)IV* |
| OH8582 | *ast-1(hd1), rol-6(su1006)II; otIs226(bas-1::gfp)IV* |
| OH8765 | *ast-1(hd1), rol-6(su1006)II; otIs221(cat-1::gfp)III* |
| OH8767 | *ast-1(rh300)II; otIs226(bas-1::gfp)IV* + anti 5-HT |
| OH8769 | *ast-1(rh300)II; otIs221(cat-1::gfp)III* |
| OH8772 | *ast-1(ot417)II; otIs221(cat-1::gfp)III* |
| OH8773 | *ast-1(rh300)II; zdIs13(tph-1::gfp)IV* |
| OH8777 | *ast-1(ot417)II; zdIs13(tph-1::gfp)IV* |
| OH904 | *otIs33(kal-1::gfp)IV* |
| OH9423 | *unc-86(n846)III; zdIs13(tph-1::gfp)IV* |
| OH9545 | *otIs287[rab-3::yfp, rol-6(su1006)]IV* |
| OH9609 | *otIs291[rab-3::gfp, rol-6(su1006)]* |
| OH9660 | *unc-86(n846)III; otIs287[rab-3::yfp, rol-6(su1006)]IV* |
| OP57 | *unc-119(ed3)III; wgIs57[sem-4::TY1::EGFP::3XFLAG(92C12)+unc-119(+)]* |
| QW122 | *zfIs6(lgc-55::gfp)II* |
| QW84 | *zfIs4(lgc-55::mCherry)* |
| RJP255 | *ynIs34(flp-19::gfp)IV* |
| RW11606 | *unc-119(tm4063)III; stIs11606[egl-18a::H1-WCHERRY + unc-119(+)]* |
| SK4013 | *zdIs13(tph-1::gfp)IV* |
| VC1503 | *egl-46(gk692)V* + anti 5-HT |
