# Supplementary material for: A transcription factor collective defines the HSN serotonergic neuron regulatory landscape
Source: eLife. 2018 Mar 22;7:e32785. doi: 10.7554/eLife.32785 (PMC5916565; doi:10.7554/eLife.32785)
Supplement: Supplementary file 4. — Related to Materials and methods. [file elife-32785-supp4.docx]

**Supplementary** **File 4. Primers**

**Primers to genotype mutant strains**

| **Gene (allele)** | **Mutation** | **Sequence** |
| --- | --- | --- |
| *ast-1(hd1)* | C>T substitution in exon 6 (hypomorph allele) | Fwd ccaagcccaagcctaagtc  Rev ggcgcacacctattttcatt |
| *ast-1(rh300)* | G>A substitution in exon 6 (hypomorph allele) |  |
| *ast-1(ot417)* | G>A substitution in exon 6 (hypomorph allele) |  |
| *unc-86(n846)* | G>A substitution disrupts splice acceptor site at intron 3 of *unc-86* longer isoform B, coinciding with the POU domain (predicted null null) | Fwd atagcctcttcagctttctccag  Rev aatctacttaggcttctgccacc |
| *unc-86(n848)* | G>A substitution disrupts splice donor site at intron 4 of *unc-86* longer isoform B, coinciding with the homeodomain (hypomorph allele) | Fwd atagcctcttcagctttctccag  Rev aatctacttaggcttctgccacc |
| *sem-4(n1971)* | G>A substitution disrupts splice donor site in exon 2 (predicted null allele) | Fwd gaagagagagtgggcggagc  Rev gccgctaaattatctgtgtaaatgg |
| *sem-4(n2654)* | C>T missense mutation in exon 6, coinciding with the second ZnF domain (hypomorph allele) | Fwd gaagagagagtgggcggagc  Rev gccgctaaattatctgtgtaaatgg |
| *hlh-3(ot354)* | C>T substitution generates early stop in exon 1 (predicted null allele) | Fwd gccctcccttatttggttgcc  Rev ctttgccttgtttccagcagc |
| *hlh-3(tm1688)* | 1244bp deletion spanning all exon 1 (predicted null allele) | External to the deletion  Fwd cgacatgttctctccgtgtttctc  Rev gctgattagaggacatcatttgtgg |
|  |  | Internal to the deletion  Fwd gccctcccttatttggttgcc  Rev ctttgccttgtttccagcagc |
| *egl-46(gk692)* | 1144 bp deletion spanning exon 1 and most of exon 2 (predicted null allele) | External to the deletion  Fwd ggaagaggagatgtgctacaagtgg  Rev gtcctcgtatttcaccttgcacagc |
|  |  | Internal to the deletion  Fwd gctcactcgctccccttcttg  Rev gctttgtctttttcgggtctatcgg |
| *egl-46(sy628)* | G>A substitution generates early stop in exon 1 (predicted null allele) | Fwd gctcactcgctccccttcttg  Rev gctttgtctttttcgggtctatcgg |
| *egl-18(n474)* | 1bp (G) deletion in exon 2 generates early stop (hypomorph allele) | Fwd ccgtttaggccgtcaatttctgg  Rev agcgactactgtctcgacaagc |
| *egl-18(n475)* | A>T substitution in exon 1 generates early stop (hypomorph allele) |  |
| *egl-18(ok290)* | 698bp deletion spanning intron 2 and exon 3 (predicted null allele) | External to the deletion  Fwd caacaatccgtgagcccacc  Rev cttcaaggatcggcaggacc |
|  |  | Internal to the deletion  Fwd ccggaagctcccaaagttgc  Rev cgatagtagagcccacacgg |

**Primers for site-directed mutagenesis (forward sequences)**

| **Construct** | **Sequence** |
| --- | --- |
| t1p26 | aggaggtgtctttgtttgtgtataccacaacaagcgatcaacacagcaaag |
| t1p31 | ttctccggatattagattaggtggcaggcggctccattg |
| t1p44 | gtatattacgtgccgaatttttgaagcaccacgccatcggat |
| t1p43 | caatcaacacagcaaagatttctctcaacctcatttcatgattttc |
| t1p60 mut1 | cgtttttttttctccggaaattagattgtgtggcaggc |
| t1p60 mut2 | gaagcaccacgccatcgtatatttaaaagaggaggtg |
| c1p61 | gtgttaagcattattctttactgaatcattgggcattctggtttccgttg |
| c1p60 | cccaccatctactgttagaaaactagcttggatccccggga |
| c1p73 mut1 | ctttactgaatcattcatcatttttttttccgttgttacccattccgccc |
| c1p73 mut2 | ccaccaaatttttcaatgttttccctgccgaaagaaaatatgaaaatatcaacg |
| c1p71 | gcccgttggtttctcttctcctgcttttaccatctactgttagaaaattg |
| c1p76 mut1 | caacaaaatataaattccagttttttttttatagcgtgtcatacagtatg |
| c1p76 mut2 | gttctttcaagtttatatcaacaaaatataaattccagtttttttttgatagcg |
| c1p63 | gaatcattcatcattctggtttcggttgttacccattcc |
| b1p72 | gcatgcctgcagccacattattggggtatttcctccaaaccac |
| b1p71 | ccagtttctatccgtttgtttggggtcaattaaattttttttttcagcgta |
| b1p65 | gaagaatacgctgaaaaaaaccccttaattgaatgcaaacaaacggatag |
| b1p78 | catctcattctcaaaccagtttctttccgtttgtttgcattcaattaa |
| b1p73 | ctcattctcaaaccagtttctatcggtttgtttgcattcaattaa |
| b1p77 | cctatccccggctttctgtttgaattccagtaacacattgatattc |
| b1p76 | ccagtaacacattgatattcttctttaacaccacattattcatgtatttcctcc |
| b1p86 mut1 | ccagaattccagtaacacatttatattcttccccaacaccac |
| b1p86 mut2 | catctcattctcaaaccagtttctttccgtttgtttgcattcaattaa |
| b1p86 mut3 | cgcaaacgttttggagaatatagacaactttaggaagtcatc |

**Probe sequences for EMSA analysis (Fwd sequences)**

| **Protein** | **Probe** | **Sequence** |
| --- | --- | --- |
| UNC-86 | tph-1 wt | gtgtctttgtttgcgcataataaaacaatcaatcaacaca |
|  | tph-1 mut | gtgtctttgtttgtgtataccacaacaagcgatcaacaca |
|  | cat-1 wt | tttactgaatcattcatcattctggtttccgttgttaccca |
|  | cat-1 mut | tttactgaatccccgggcattctggtttccgttgttaccca |
|  | bas-1wt | aaaccagtttctatccgtttgtttgcattcaattaaattttttttttcagcgtattc |
|  | bas-1 mut | aaaccagtttctatccgtttgtttggggtcaattaaattttttttttcagcgtattc |
| AST-1 | cat-1 wt | tttactgaatcattcatcattctggtttccgttgttaccca |
|  | cat-1 mut | tttactgaatcattcatcattctggttgttaccca |
|  | bas-1 wt | ctcattctcaaaccagtttctatccgtttgtttgcattcaattaaatt |
|  | bas-1 mut | ctcattctcaaaccagtttctttgtttgcattcaattaaatt |
| EGL-18 | cat-1 wt | gtttatatcaacaaaagataaattccagtttttttttgatagcgtgtcatacag |
|  | cat-1 mut | gtttatatcaacaaaatataaattccagttttttttttatagcgtgtcatacag |

**Primers for CRISPR-Cas9 mediated GFP knock in**

| **Use** | **Sequence** |
| --- | --- |
| Amplify *ast-1* homology arm 5’. External. | Fwd tgctcctgatttctcatcgtgg  Rev tcgataaagagggaatgctcg |
| Amplify *ast-1* homology arm 5’. Nested. | Fwd acgttgtaaaacgacggccagtcgccggcacgatctctgaatattgccggg  Rev catcgatgctcctgaggctcccgatgctcctcgataaagagggaatgctcgtg |
| Amplify *ast-1* homology arm 3’. External. | Fwd tagtcaccccccataattcct  Rev gcgagacccaccaaattgattc |
| Amplify *ast-1* homology arm 3’. Nested. | Fwd cgtgattacaaggatgacgatgacaagagatagtcaccccccataattcctcc  Rev ggaaacagctatgaccatgttatcgatttcattgattccgtgcgcctttg |
| *ast-1* sgRNA | Fwd ctcctattgcgagatgtcttgggggtgactatcgataaagagttttagagctagaaatagcaag  Rev cttgctatttctagctctaaaactctttatcgatagtcacccccaagacatctcgcaataggag |
| Amplify *hlh-3* homology arm 5’. External. | Fwd aaatttgcggtttgattataggg  Rev gatgcagaagatgatgagctttc |
| Amplify *hlh-3* homology arm 5’. Nested. | Fwd acgttgtaaaacgacggccagtcgccggcagcaatgttttagaataagctgcc  Rev catcgatgctcctgaggctcccgatgctccataagtttctgtatgcgaataaaagctatgatgatcaccagaagttgatgcagaagatgatgagctttcttg |
| Amplify *hlh-3* homology arm 3’. External. | Fwd taatctgttaagttgtaccatattttc  Rev actgaaagacaacatttgttaactg |
| Amplify *hlh-3* homology arm 3’. Nested. | Fwd cgtgattacaaggatgacgatgacaagagataatctgttaagttgtaccatattttctg  Rev ggaaacagctatgaccatgttatcgatttctgattgaagatcttatcaaaatgtg |
| *hlh-3* sgRNA | Fwd ctcctattgcgagatgtcttggctatgatgatcaccagaaggttttagagctagaaatagcaag  Rev cttgctatttctagctctaaaaccttctggtgatcatcatagccaagacatctcgcaataggag |

**Primers for cloning**

| **Cloned region** | **Sequence** |
| --- | --- |
| *ast-1* cDNA  Primers A* | Fwd gagagaggtaccatgcaagtcgtctcgtcagc |
|  | Rev gagagagaattcctatcgataaagagggaatgctcgt |
| *ast-1* cDNA  Primers B* | Fwd gagagaggtaccggtagaaaaaatgatgcaagtcgtctcgtcagcc |
|  | Rev gagagagaattcctatcgataaagagggaatg |
| *hlh-3* cDNA  Primers A* | Fwd gagagaggtaccatgaccgcatccacctcc |
|  | Rev gagagagaattcttaataagtttctgtatgcgaataaaagctatg |
| *hlh-3* cDNA  Primers B* | Fwd gagagaggtaccatgaccgcatccacctc |
|  | Rev gagagagaattcttaataagtttctgtatgcg |
| *hlh-3* cDNA  Primers C* | Fwd gagagaggtaccatgaccgcatccacctc |
|  | Rev gagagaggtaccttaataagtttctgtatgcg |
| *egl-46* cDNA | Fwd gagagaggtaccatggtgcctatgaatg |
|  | Rev gagagagaattcttacattgttggaataac |
| Pet1 cDNA | Fwd gagagaggtaccatgagacagagcggcacctc |
|  | Rev gagagagaattcctagtgataatgaccccccaag |
| Ascl1 cDNA | Fwd gagagaggtaccatggagagctctggcaag |
|  | Rev gagagagaattctcagaaccagttggtaaagtcc |
| Insm1 cDNA | Fwd gagagaggtaccatgccacggggatttc |
|  | Rev gagagagaattctcaacaagcgggc |
| Sall2 cDNA | Fwd gagagacccgggatgtctcggcgaaag |
|  | Rev gagagagtatactcatggcatggtgg |
| Gata2 cDNA | Fwd acattttcaggaggacccttggagggtaccatggaggtggcgcctgagcag |
|  | Rev atggtagcgaccggcgctcagttggaattcctagcccatggcagtcaccatgc |
| *bas-1* prom | Fwd aaaggatccggaaatggcaacatcttagac |
|  | Rev tttggatccccgaactactactgaaagttc |
| *cat-4* prom | Fwd gagagactgcaggtgagtgaaggtctatatcattc |
|  | Rev gagagaggatccaatggatagacttcatcatcttttc |
| *kal-1* prom | Fwd gagagactgcagatttcgtatttggagc |
|  | Rev gagagaggatcccatgtgctgtaagag |

* Primers A were used to amplify the cDNAs used in HSN early maturation experiments, while primers B were used to amplify the same cDNAs but for their use in mutant HSN rescue experiments, and primers C were used to amplify *hlh-3* cDNA used in heat shock mediated mutant rescue.

**Primers for putative enhancers of the HSN**

| **Gene** | **Gene ID** | **Sequence** |
| --- | --- | --- |
| *aak-2* | T01C8.1 | Fwd tcatcggaccaacttcccc  Rev cccactaaattttcctgtagtttcagc |
| *abts-4* | R03E9.3 | Fwd tcctcactctgtcatttgaatgttctc  Rev aactgttatcatatctcacacataatttacgc |
| *acr-24* | Y73F8A.30 | Fwd aatctgatcaattctaaactatttttttcaacacc  Rev aggtttgaaacatttttttgaaacaaaatttgaag |
| *ast-1* | T08H4.3 | Fwd ggtaaattcccaaattttggccaaac  Rev tcaattgcatagaacaattaggttatgcc |
| *bam-2* | Y71F9AR.1 | Fwd ggaacaagtcaaggtgttcatagaaatag  Rev acattcattggacgcgtacaatttc |
| *C16B8.4* | C16B8.4 | Fwd ggatgtggaacatgaatccgattg  Rev ggggtagaatgggtgaaaaaaaattgt |
| *C53B4.4* | C53B4.4 | Fwd gcttcaggaagcgaacaagc,  Rev ggagcacacgtttctaagatggg |
| *ckr-2* | Y39A3B.5 | Fwd ctaagatttgctcctcttattttagccg  Rev catcttcttcttcttccccttcttcta |
| *daf-38* | Y105C5A.23 | Fwd catagaaatttctgatgatgagcctcg  Rev tgccttaaaaacatagtgaaacgttagg |
| *dgn-1* | T21B6.1 | Fwd gtgaaaaaagtctttcatcgcaaaacc  Rev gcaacacgcatacacacacaa |
| *f16g10.5* | F16G10.5 | Fwd cctaactggatgactcagtaccaaaaag  Rev atgaataccttcttcaatccaacagaaag |
| *F32D8.10* | F32D8.10 | Fwd gaattttcatgattcatcttgaaaatcccc  Rev cctgggtagttgatcttctacttgaag |
| *F37A8.5* | F37A8.5 | Fwd catgtgatgtgagaattcccaatgg  Rev tcgtcatgaatttatttatatattggtacaagcg |
| *F56A4.2* | Y19D10A.7 | Fwd gaaagagctaccactaaacgaaacatg  Rev agttttttaaaaatgatttttgggaaattggaaaa |
| *flp-27* | C25H3.5 | Fwd gcaaatcgacaatttgccgaaaatg  Rev acctctgtgaaagccacgc |
| *fut-1* | K08F8.3 | Fwd ttttttttgaaaacatcgtcaccgc  Rev tttcctgttttacagtgattcacataagc |
| *gab-1* | ZC482.1 | Fwd ccgaggatcagttatgtgaagagtattg  Rev cagacacgaagaaattcattacgaaaatcg |
| *gipc-2* | F44D12.4 | Fwd ggcgtcaactaacaatgacgtg  Rev atgtatagatttttgcctcaaaatccagg |
| *glb-20* | R01E6.6 | Fwd cggagagtgagaaagagagagagg  Rev agggtaaaaagtattcaaaactcaacagg |
| *irld-62* | ZK1037.1 | Fwd gtaaaaatcctaaacattaggatagttttttgatgtg  Rev caattgaacaaactataatatttttcatgaaaaaatactttaaaacc |
| *kal-1* | K03D10.1 | Fwd tcgctaaaaaaatctcttgaagtctgc  Rev aaacatctgtactagtccgggttc |
| *kcc-1* | R13A1.2 | Fwd cagaaatccatccactgaaacagt  Rev caaaaacccagcagagcgtg |
| *kcc-2* | K03D10.1 | Fwd atttaacaacattgcaaaacagaagaagtc  Rev cagaattggtattaataacgggatgaaagg |
| *kel-8* | W02G9.2 | Fwd atcgtaaacataaacaatgcacccg  Rev gggtccaaaaaaatcgtatttttgcg |
| *klp-7* | K11D9.1 | Fwd gtctaattgcctctgatatgttttgacc  Rev gatgttatgaaatggcgagtactgc |
| *lgc-49* | K10D6.1 | Fwd atgaaccctctttcacttttggc  Rev cgaacaaggaacatgtctgtaatgtg |
| *lurp-2* | F25H9.1 | Fwd acatcgcagcgacaaagtttttg  Rev ctcgaacgttagagcctccttg |
| *mec-10* | F16F9.5 | Fwd attgattgcactaataatccactggc  Rev tcctgttttagctcaaaatacgtgc |
| *mgl-2* | F45H11.4 | Fwd agtgtaatgaaccaaaaaataggcgg  Rev acaaaaaagcgttccaattcctcg |
| *npr-1* | C39E6.6 | Fwd aatagctctgaatcattctaaaacgcc  Rev attgaatttggaacgagtaatgtgtagg |
| *npr-3* | C10C6.2 | Fwd acaaatcaaaaccgcaaaaaacagg  Rev acaagcgatatggcatggacc |
| *pan-1* | M88.6 | Fwd cgaaagtacaacaatgattcctcatagg  Rev gatcgtaaaatcttaattcacagaagctcg |
| *pde-3* | E01F3.1 | Fwd cgtacaatttttttttggaaaaatcaaaaaaaattaagc  Rev actgtgactttttgaaatttttttcccg |
| *plep-1* | Y52E8A.4 | Fwd ccaatacatttccagttcaaaaaagttttttttaatac  Rev tctgaatattttttgtgaaatattgaaaaaactcttcg |
| *sem-4* | C32E12.5 | Fwd aactcttaatgttttgttgcgaccc  Rev ggtcgtaaaaatcgcaacaaaccg |
| *shl-1* | Y73B6BL.19 | Fwd gttcgaaaattttggaattgactaattttatccg  Rev gctagcacctattgagcaggaa |
| *slc-28.1* | F27E11.1 | Fwd ttcctagcggataaattcaaagtttttttaatg  Rev agcttttggtaactgaaatcagatttttttc |
| *snt-1* | F31E8.2 | Fwd aagaaaaggttatgcaacaaactggg  Rev gcgagaaccagcagaataaatacg |
| *sprr-1* | R03A10.6 | Fwd cgtcattgtcttggtttgtctcg  Rev tcttgggaaaaaatatgttaagaaggtgc |
| *stg-1* | C18D1.4 | Fwd gaaactttcaaattagctgaatcagttgattttc  Rev ttttaaaacaaatatgacggggcttttcg |
| *sto-5* | F41G4.3 | Fwd gtcactccgaggttctggc  Rev agaagaagaatgtacagatatagtgcgc |
| *tiam-1* | C11D9.1 | Fwd actactcgagttgagtgtgttgc  Rev ctgttatggcaagttgaaactggag |
| *tkr-2* | C49A9.7 | Fwd ttaattgctacttaatcgagaagttcgaac  Rev agtgaaaaaattatttgaatggccacatc |
| *tol-1* | C07F11.1 | Fwd ccttgttaccttgactatcgggaa  Rev cctaattagtagtcacgagaagagcag |
| *tub-1* | F10B5.4 | Fwd gctaaaaattatacattcatttatgttg  Rev gattacctggaaactttgaatagtttttgaac |
| *twk-17* | C44E12.3 | Fwd ggtttacagctttgaagactagtaagc  Rev tcactgatgtctagacttaagcaagattc |
| *tyra-3* | M03F4.3 | Fwd agtgcgtgtatgtctctaacatctaag  Rev gaaatctagttatcggttaggttaatttcgg |
| *unc-32* | ZK637.8 | Fwd ctcatgattcatttctctccttattggc  Rev cgcttctttcgagagaaaacatttaaga |
| *unc-7* | R07D5.1 | Fwd gactgagctatcctgcctgc  Rev tcaatgcaagaaagacacgcg |
