# Supplementary material for: A transcription factor collective defines the HSN serotonergic neuron regulatory landscape
Source: eLife. 2018 Mar 22;7:e32785. doi: 10.7554/eLife.32785 (PMC5916565; doi:10.7554/eLife.32785)
Supplement: Transparent reporting form [file elife-32785-transrepform.pdf]

eLIFE
1st Floor
24 Hills Road
Cambridge CB2 1JP, UK
P 01223 855340
W elifesciences.org
T @elife

eLife's transparent reporting form

We encourage authors to provide detailed information *within their submission* to facilitate the interpretation and replication of experiments. Authors can upload supporting documentation to indicate the use of appropriate reporting guidelines for health-related research (see EQUATOR Network), life science research (see the BioSharing Information Resource), or the ARRIVE guidelines for reporting work involving animal research. Where applicable, authors should refer to any relevant reporting standards documents in this form.

If you have any questions, please consult our Journal Policies and/or contact us: editorial@elifesciences.org.

Sample-size estimation

- You should state whether an appropriate sample size was computed when the study was being designed
- You should state the statistical method of sample size computation and any required assumptions
- If no explicit power analysis was used, you should describe how you decided what sample (replicate) size (number) to use

Please outline where this information can be found within the submission (e.g., sections or figure legends), or explain why this information doesn't apply to your submission:

No sample size estimation was computed *a priori* for expression analysis in *C. elegans*. We selected minimum sample sizes based on what is customary in the field and on our previous experience (e.g. Flames and Hobert, 2009; Doitsidou et al, 2013; Patel and Hobert, 2017).
Detailed information regarding sampling size and replicates can be found in Supplementary Tables 1 and 3, methods and supplementary methods. As a summary, for reporter expression analysis in mutant backgrounds, serotonin immunostaining, transcription factor rescues and RNAi maintenance experiments a minimum of 100 cells was analyzed per replicate (corresponding approximately to 50 animals). For transcription factor developmental analysis, a minimum of 60 cells was scored per developmental time point, except for embryos where 40 cells were scored in some cases (corresponding approximately to 20 to 30 animals) . For *cis*-regulatory analysis and *de novo* discovery of HSN expressed genes expression was assessed in a minimum of 60 cells (corresponding approximately to 20 to 30 animals) in each transgenic line, which account for biological replicates.

Replicates

- You should report how often each experiment was performed
- You should include a definition of biological versus technical replication
- The data obtained should be provided and sufficient information should be provided to indicate the number of independent biological and/or technical replicates
- If you encountered any outliers, you should describe how these were handled
- Criteria for exclusion/inclusion of data should be clearly stated

eLife Sciences Publications, Ltd is a limited liability non-profit non-stock corporation incorporated in the State of Delaware, USA, with company number 5030732, and is registered in the UK with company number FC030576 and branch number BR015634 at the address 1st Floor, 24 Hills Road, Cambridge CB2 1JP | August 2014

- High-throughput sequence data should be uploaded before submission, with a private link for reviewers provided (these are available from both GEO and ArrayExpress)

Please outline where this information can be found within the submission (e.g., sections or figure legends), or explain why this information doesn't apply to your submission:

Expression of each extrachromosomal array was assessed in three independent *C. elegans* transgenic lines as a rule (these account for biological replicates since arrays are formed independently in the germ line of different worms injected with DNA). In some cases, we were unable to obtain three lines and thus only one or two of them were analyzed.

In the case of *cis*-regulatory analysis, whenever there was high variation between lines, expression was evaluated in up to six independent lines (data in Supplementary Figures 2 and 3). Data for *de novo* discovery of promoters active in HSN can be found in Supplementary Table 3. In the case of transcription factor rescues only one or two lines were used because they were difficult to obtain (data in Supplementary Table 1).

RNAi experiments were performed twice. Here, repetitions account also for biological replicates (data in Supplementary Table 1).

Serotonin immunostaining in *C. elegans* was also performed twice, with the exception of *sem-4* mutant immunostaining that was only performed once. In this case, repetitions are considered technical replicates (data in Supplementary Table 1).

Statistical reporting

- Statistical analysis methods should be described and justified
- Raw data should be presented in figures whenever informative to do so (typically when N per group is less than 10)
- For each experiment, you should identify the statistical tests used, exact values of N, definitions of center, methods of multiple test correction, and dispersion and precision measures (e.g., mean, median, SD, SEM, confidence intervals; and, for the major substantive results, a measure of effect size (e.g., Pearson's r, Cohen's d)
- Report exact p-values wherever possible alongside the summary statistics and 95% confidence intervals. These should be reported for all key questions and not only when the p-value is less than 0.05.

Please outline where this information can be found within the submission (e.g., sections or figure legends), or explain why this information doesn't apply to your submission:

For all comparisons of reporter expression in HSN between different genotypes and/or lines, two tailed Fisher's exact test was used, as stated in the methods section (Statistical analysis for HSN scorings). For each experiment, exact p-values are indicated in Supplementary Table 1 and * = p-value< 0,05 is indicated in the figure legend.

For evaluation of HSN regulatory signature enrichment in HSN expressed genes and hierarchical clustering, detailed information can be found in the methods section, (Bioinformatics analysis) and in the corresponding section of the supplementary methods.

(For large datasets, or papers with a very large number of statistical tests, you may upload a single table file with tests, Ns, etc., with reference to sections in the manuscript.)

Group allocation

- Indicate how samples were allocated into experimental groups (in the case of clinical studies, please specify allocation to treatment method); if randomization was used, please also state if restricted randomization was applied
- Indicate if masking was used during group allocation, data collection and/or data analysis

Please outline where this information can be found within the submission (e.g., sections or figure legends), or explain why this information doesn't apply to your submission:

Does not apply

Additional data files ("source data")

- We encourage you to upload relevant additional data files, such as numerical data that are represented as a graph in a figure, or as a summary table
- Where provided, these should be in the most useful format, and they can be uploaded as "Source data" files linked to a main figure or table
- Include model definition files including the full list of parameters used
- Include code used for data analysis (e.g., R, MatLab)
- Avoid stating that data files are "available upon request"

eLife Sciences Publications, Ltd is a limited liability non-profit non-stock corporation incorporated in the State of Delaware, USA, with company number 5030732, and is registered in the UK with company number FC030576 and branch number BR015634 at the address 1st Floor, 24 Hills Road, Cambridge CB2 1JP | August 2014

Please indicate the figures or tables for which source data files have been provided:

In Supplementary Table 1 all scorings in figures 1, 2, 5, 6 and 7 are included as percentage of expression, standard error of the proportion and p-value.
In Supplementary Table 2 a complete list of genes used in HSN regulatory signature bioinformatics analysis is included.
In Supplementary Table 3 the list of genes with conserved HSN regulatory signature used for *de novo* expression identification in the HSN, together with expression analysis data is included.
In Supplementary Table 4 all strains used in this work are included.
In Supplementary Table 5 a complete list of the primers used in this work is included.
In the R code HSN regulatory signature analysis supplementary file we include R code and bash pipeline for the evaluation of HSN regulatory signature enrichment in HSN expressed genes.
